# REDUCING AGEISM IN THE LGBTQ+ COMMUNITY THROUGH INTERGENERATIONAL CONNECTION

**DOI:** 10.1093/geroni/igac059.036

**Published:** 2022-12-20

**Authors:** Carrie Andreoletti, Christina Barmon, Andrea June, Michael Bartone

**Affiliations:** Central Connecticut State University, New Britain, Connecticut, United States; Central Connecticut State University, New Britain, Connecticut, United States; Central Connecticut State University, New Britain, Connecticut, United States; Central Connecticut State University, New Britain, Connecticut, United States

## Abstract

Intergenerational interaction has the potential to reduce ageism and increase feelings of generativity in both younger and older adults. To expand aging education beyond our aging and gerontology classes, we collaborated with our campus LGBT Center and community partners to host several intergenerational conversations between younger and older LGBTQ+ adults and allies. The goal was to foster connection across the generations in the LGBTQ+ community through discussions of topics of mutual interest (e.g., ageism, identity & language). Participants in the LGBTQ+ conversations reported that they valued the opportunity to talk with members of the community from different generations and that the conversations changed their views of one another in a positive way. We will discuss the strengths and challenges of our program, ideas for future programs and research, and suggestions for integrating discussion of LGBTQ+ aging into the classroom.

